# Quality Characteristics of Reduced-Fat Emulsified Sausages Made with Yeast Mannoprotein Enzymatically Prepared with a β-1,6-glucanase

**DOI:** 10.3390/foods12132486

**Published:** 2023-06-26

**Authors:** Lingli Zhong, Xiangrui Guo, Huizhen Xue, Yan Qiao, Dongmei Mao, Xianfeng Ye, Zhongli Cui, Zhoukun Li, Gang Hu, Yan Huang

**Affiliations:** 1 Key Laboratory of Agricultural Environmental Microbiology, Ministry of Agriculture and Rural Affairs, College of Life Sciences, Nanjing Agricultural University, Nanjing 210095, China; 2022216012@stu.njau.edu.cn (L.Z.); 2021116058@stu.njau.edu.cn (X.G.); 2022816121@stu.njau.edu.cn (H.X.); maodongmei@njau.edu.cn (D.M.); yxf@njau.edu.cn (X.Y.); czl@njau.edu.cn (Z.C.); zkl@njau.edu.cn (Z.L.); 2 West China School of Pharmacy, Sichuan University, Chengdu 610064, China; 2016216018@njau.edu.cn; 3 Laboratory Center of Life Science, Nanjing Agricultural University, Nanjing 210095, China

**Keywords:** emulsified sausage, emulsion, mannoprotein MP112, nutritional composition

## Abstract

Mannoproteins, as yeast polysaccharides, have been utilized in food the industry as dietary fibers, emulsifying agents or fat replacers. Mannoprotein MP112, produced from yeast by enzymatic hydrolysis of myxobacterial β-1,6-glucanase GluM, exhibits excellent emulsifying properties in emulsion preparation. In this study, we aimed to examine the application of stable emulsion with the addition of mannoprotein MP112 (MP112 emulsion) to reduce the fat content of sausages. The addition of MP112 emulsion in emulsified sausages significantly reduced the fat content and increased the moisture and protein contents of emulsified sausages without the expense of their good sensory quality. Moreover, the textural properties of sausages were markedly improved with the higher hardness, chewiness and cohesiveness, especially in the 50–75% replacement ratio of MP112 emulsion. On the other hand, MP112 emulsion replacement of animal fat markedly improved the nutritional composition of emulsified sausages; they displayed a higher PUFA/SFA ratio and lower n-6/n-3 ratio due to their saturated fatty acids being replaced by poly-unsaturated fatty acids. Meanwhile, the oxidative stability of sausages was improved linearly, corresponding to the increased replacement ratio of MP112 emulsion. Our results show that mannoprotein-based emulsions could be used as potential fat alternatives in developing reduced-fat meat products.

## 1. Introduction

Sausages became one of the most favored meat products in the world thanks to their nutritional value and attractive sensory profiles. However, emulsified sausages generally contain high fat contents (20–30%), which are associated with obesity, high blood pressure, cardiovascular, non-alcoholic fatty liver diseases and other chronic diseases [1,2]. The recommended dosage of fat intake in the diet is 15–30%, and the healthy intake of saturated fatty acids should be less than 10% [3]. Based on the recent increase in demand for healthier diets, the production of reduced-fat sausages with a healthier nutritional composition has become a hot topic in the meat products industry. However, reducing the fat content in meat products without considering composition can lead to a decrease in the flavor and quality of meat products with poor texture parameters [4]. Therefore, to meet current consumer needs, developing suitable fat replacers to replace animal fat in meat products without sacrificing sensory and textural quality is a major challenge for meat industries.

At present, fat replacers are mainly divided into the three categories: fat-based, carbohydrate-based, and protein-based products [5]. Various types of plant-based oil emulsions have been applied as fat replacers in processed reduced-fat meat products. Meanwhile, plant-based oils, such as soy, olive, peanut, tea seed, coconut, palm and maize oils, exhibit a positive impact on nutritional composition by reducing cholesterol content and improving fatty acid composition [6]. However, the applications of the oil-in-water emulsions are limited in food systems due to physical instability, soft texture and oxidation problems [7]. To overcome these problems, several strategies have been developed, including the utilization of proteins (soy proteins, sodium caseinate, whey proteins, corn germ protein), carbohydrates (starches, konjac glucomannan, cellulose, inulin, oat fiber, polysaccharide), hydrocolloids (xanthan, locust bean gum) and even natural antioxidants (curcumin, carotenes, annatto bixin, capsorubin) [8,9,10]. Zhang et al. [11] used water-soluble β-glucan to make low-fat sausages with the presence of peanut protein isolate or soy protein isolate, which significantly improved the water-holding capability and increased the hardness, chewiness and cohesiveness of prepared sausages. Li et al. [12] reported that the utilization of stabilized emulsion containing zein/carboxymethyl dextrin and carrageenan could enhanced the texture and improved the oxidative stability of sausages. Therefore, composite-based emulsions display better functionality than those with a single matrix, such as protein–polysaccharide composite emulsion, in which polysaccharides and proteins could be combined via the complexation process to improve the texture and network structures [13].

Mannoproteins are glycoproteins mainly isolated from yeast, which consist of a backbone formed by mannose units linked by α-(1-6) bonds and side chains composed of oligosaccharides [14]. Mannoproteins mainly contain 80–90% of carbohydrates and 5–20% of protein, and the contained essential amino acids are higher than those of plant proteins [15,16]. Mannoproteins have found great applications in the wine making industry for their techno-functional properties, such as their reduction of free ochratoxins and phenolic compounds, their growth promotion of malolactic bacteria and their limitation of tartrate salt crystallization [17]. Due to their amphiphilic nature, i.e., their consisting of hydrophobic proteins and hydrophilic mannose polymers, mannoproteins have been regarded as effective bio-emulsifiers, widely used in several food applications, such as French salad dressing [18]. Ashraf et al. [19] reported that mannoproteins exhibited an effective emulsifying property compared to whey protein concentrate under a broad range of conditions. Furthermore, mannoproteins are regarded as a novel prebiotic food ingredient, and recent studies reported that mannoproteins show anticancer, antioxidant, antimicrobial and prebiotic properties [20,21], which exhibit positive effects on obesity and gut microbiota dysbiosis induced by high-fat diets [22]. These results indicate that mannoproteins present great potential in food applications due to their excellent emulsifying and prebiotic properties.

In our previous study, mannoprotein MP112 was produced based on a gentle and environmentally friendly approach from baker’s yeast using a myxobacterial β-1,6-glucanase GluM, which exhibited high purity of 90% and molecular weight of 112.7 kDa [23]. The starting materials and isolation methods determine the composition and structure of mannoproteins, resulting in different functional properties [24,25]. Mannoprotein MP112 exhibited different chemical and morphological characterizations, with higher content of hydrophilic residues and 4.14% methionine, and had favorable emulsifying and emulsion-stabilizing properties. The application of MP112-stabilized emulsion (6% mannoprotein MP112 content in emulsion formulation) in myofibrillar protein composite gel could form a fine-stranded gel network and thereby improve the strength and water holding ability of composite gels.

Currently, whether mannoprotein-based emulsion could be applied as a fat replacer in reduced-fat sausage production is unknow. Therefore, the goal of this study is to use the MP112-stabilized emulsion to reduce fat content in sausages and evaluate its effect on the nutritional composition, oxidative stability and textural and sensory quality of emulsified sausages.

## 2. Materials and Methods

### 2.1. Preparation of MP112 Emulsion

According to the method reported by Qiao et al. [26], mannoprotein MP112 was prepared from baker’s yeast by the β-1,6-glucanase GluM enzymolysis (Figure S1). According to the published emulsion formulation in our previous studies [23], the emulsion with the addition of mannoprotein MP112 (MP112 emulsion) was prepared as described below. A total of 5 g sodium caseinate was dissolved in 50 mL deionized water, followed by the addition of same mass ratio olive oil blend (Betis, Torres Y Ribelles S.A.), which contains 20% olive oil and 80% sunflower oil. Then, these mixtures were vortexed to homogeneity in ice water bath using homogeneous mixer (Ultra Turrax T-25 Basic, IKA Co., Staufen, Germany). A total of 6 g mannoprotein and appropriate deionized water was added into the prepared solutions with vortexing at 15,000 rpm three times (1 min each time at an interval of 30 s) to a constant volume of 100 mL. The mass fraction of the oil phase, sodium caseinate and mannoprotein in emulsion was 50%, 5% and 6% (*w/v*), respectively. The preparation of MP112 emulsion was replicated thrice.

### 2.2. Reduced-Fat Emulsified Sausage Making

The ratio of lean fat in traditional emulsified sausage was 70:30 (wt/wt). A total of 24% pork backfat was added in control samples (ME0), while in other formulations, pork backfat was substituted with MP112 emulsion at levels of 25% (ME25), 50% (ME50), 75% (ME75) and 100% (ME100). Reduced-fat emulsified sausages with different replacement ratio of MP112 emulsion were prepared according to the designed formulations shown in Table 1. Pork leg muscle (all visible fat and connective tissue were trimmed) and back fat were purchased from a local commercial meat processing company (Sushi Group, Nanjing, China); the obtained pigs were slaughtered according to the requirements of National Standards of China “Pig Slaughter and Quarantine Regulations”. The required amount of pork leg muscle, salt, sugar, phosphate, and pepper were added and chopped at high speed with 1/3 iced water for 1.5 min using a vacuum chopper (BZBJ-40, Hangzhou Aibo Technology Engineering Ltd., Hangzhou, China). The pork back fat was added and chopped for 1.5 min with 1/3 the iced water, followed by addition of the MP112 emulsion with the rest of the iced water chopping for 1.5 min. The temperature of all meat batter samples was kept below 16 °C.

The mixtures were stuffed into hog gut casings (24 mm diameter) using a sausage stuffer (EB-12, Mainca UK Ltd., Berks, PA, USA) to form sausages (60 g, 15 cm long), followed by cooking at low temperature of 80 °C in a water bath for 40 min with an internal temperature of around 74 °C. Sausages were then taken and cooled to room temperature with plastic bag packaging. The production process of sausages was replicated thrice. For subsequent analysis, sausages of each treatment were kept at 4 °C without access to light and randomly taken within a week of sausage production.

### 2.3. Physicochemical Analysis

Color measurements: After equilibration to room temperature for 30 min, color parameters were assessed on the fresh surface of 2 cm thick sausage slices at room temperature using a CR-400 colorimeter (Minolta Camera Co., Osaka, Japan) and Illuminate C, calibrated with a white plate. Color parameters from six replicate samples (L*, lightness; a*, redness; b*, yellowness) were recorded using the average values of four measurements taken from different locations on each sausage slice.

Cooking loss and proximate analysis: The cooking loss of emulsified sausages from different treatments was determined by recording the weight difference between the uncooked and cooked sausages, which was expressed as a percentage of initial weight. Each sausage sample weight was measured after equilibration to room temperature.

Crude protein, fat, moisture and ash content were determined by referring to the AOAC official methods [27]. The moisture of sample content was determined by drying at 105 °C in an oven until a constant weight, and the crude protein content of the samples was determined using the Kjeldahl method (Kjeltec™ 8000, Foss Co., Hillerod, Denmark). The conversion factor of total nitrogen converted to the crude protein was designed as 6.25. Crude fat content was determined using a Soxhlet system (GY-ZFCDY-6P Soxhlet extractor, Shanghai Guiyong Co., Shanghai, China) after ether extraction. Ash content was determined by burning the samples at 550 °C in a muffle furnace for 8 h.

Texture profile analysis: After equilibration to room temperature for 30 min, sausages were cut into cylindrical segments with flat cut surfaces (diameter 24 mm, height 10 mm) and subjected to texture profile analysis using TA. XT PlusC (Stable Micro Systems Ltd., Goldaming, UK), equipped with a 50 mm diameter cylindrical probe (P/50). The parameters are used as follows: the test speed, 2 mm/s; compression rate, 50%; the return speed, 5 mm/s. Texture parameters (hardness, springiness, cohesiveness and chewiness) were recorded with six repetitions.

### 2.4. Fatty Acid Analysis

The fat of emulsified sausages was extracted with a chloroform-methanol (2:1 *v/v*) solution as previously described [28]. After saponification treatment in 0.5 mol/L sodium hydroxide methanol solution at 60 °C for 30 min, fatty acids were then converted into methyl esters with 14% boron trifluoride methanol solution. Then, fatty acid methyl esters were dissolved in 1.5 mL of hexane. Fatty acid composition was analyzed by a gas chromatograph (Trace GC Ultra, Thermo Fisher Scientific Inc., Cleveland, OH, USA) and a 100-m capillary column (SP 2560, Supelco, Bellefonte, PA, USA) with a split ratio of 100:1. The temperature program was designed as follows: ramped oven temperature at 140 °C for 2 min, followed by increasing to 225 °C at 5 °C/min and maintained at 225 °C for 17 min; the inlet temperature and the detector temperature was 240 °C. The injection volume was 1 μL with nitrogen as carrier gas. The fatty acid methyl esters of samples were identified by comparing the retention times to standard fatty acid methyl ester mixtures (Supelco 37 Component FAME mix, Sigma, Bellefonte, PA, USA).

### 2.5. Lipid Oxidation Measurement

For analysis of the evolution of lipid oxidation, emulsified sausage samples were implemented in triplicate from each batch from 0, 3, and 7 days stored at 4 °C. Lipid oxidation was analyzed by the 2-thiobarbituric acid-reactive substances (TBARS) method with minor modifications [29]. Samples (±5 g each) were minced by the vacuum chopper and vortex-mixed with 25 mL of 7.5% TCA solution containing 0.1% EDTA. A total of 2 mL of the supernatant was aspirated, followed by mixing with 2 mL 0.02 mol/L MDA and heating at 95 °C for 30 min. After cooling to room temperature, the absorbance of the clear pink layer was recorded at 532 nm using a spectrophotometer UV Mini 1240 (Shimadzu Corp., Kyoto, Japan), and the treatments without the sample were used as control. The result was expressed in milligram of malondialdehyde (MDA) per kg of meat.

### 2.6. Sensory Evaluation

Sensory evaluation was performed by 12 trained panelists from Nanjing Agricultural University, consisting of 5 males and 7 females (age range 23 to 45) in a quiet room under a mixture of fluorescent and natural light without communication. Sausage samples were warmed on the plate after being cut into pieces and encoded with random numbers. Each panelist was required to use distilled water for gargling between each sample evaluation. A 9-point hedonic scale was used to assessed the appearance, flavor, texture, juiciness and overall acceptability of sausage samples from different treatments with a numerical value ranging from 1 = dislike extremely to 9 = like extremely [30]. After deducting the outliers, the sensory score was averaged.

### 2.7. Statistical Analysis

Data were analyzed using the SPSS version 17.0 (SPSS Inc., Chicago, IL, USA) by one-way ANOVA. ANOVA mean comparisons were performed in terms of the Duncan’s multiple-range test at the significance level of *p* < 0.05. The correlations among variables were determined by two-tailed Pearson’s correlation coefficient.

## 3. Results and Discussion

### 3.1. Color Analysis

As the first qualitative criterion of meat products, color plays a critical role in consumer’s perception and product acceptability. The results of the color of emulsified sausages prepared with different replacement ratios of MP112 emulsion are analyzed. As presented in Table 2, the L* value of reduced-fat emulsified sausages with increasing degrees of emulsifier substitution significantly increased, ranging from 76.54 to 78.56 (*p* < 0.05). The a* and b* values of sausage samples with different treatments ranged within 2.07~2.46 and 1.07~1.49, with a change of below one unit. Compared with the control treatment (ME0), the a* and b* values decreased significantly in ME75 and ME100 samples (*p* < 0.05).

Animal fat replaced by vegetable oils could decrease the value of redness but enhance lightness in emulsified meat products such as camellia oil, olive oil and canola oil. Using vegetable oils as fat replacers in sausages exhibited better distribution than the animal fat tissue, with an increase in the area of fat particles which generated more light reflection, whereas the redness of sausages might decrease due to the change in the concentration of myoglobin. Furthermore, typically, yellowness in sausages corresponds to the composition of fatty acids and the degree of lipid oxidation; thus, the use of vegetable oils to replace animal fat sometimes increased the yellowness of sausages [31,32,33,34,35]. The decreased b* value of emulsified sausages with the addition of MP112 emulsion might be related to the changes of fatty acids’ composition and their lipid oxidation in sausages. However, the differences in the a* and b* values of emulsified sausages with different treatments were really small (below 1 unit), which could not be detected by the human eye [36].

### 3.2. Cooking Loss and Proximate Composition

The cooking loss and proximate analysis of emulsified sausages with different treatment are shown in Table 3. The cooking loss of sausage samples with different treatment ranged within 1.88%~2.18%. As the degree of substitution of MP112 emulsion increased above 25%, sausage samples with MP112 emulsion presented less cooking loss than the control. Choi et al. [37] also reported similar results, i.e., that the replacement of pork fat by sunflower seed oils and dietary fiber in frankfurter formulation could reduce the cooking loss of reduced-fat frankfurters. According to Kairam et al. [38], the ability to control the cooking loss can be related to the ability of the protein matrix to immobilize water and fat molecules. The addition of MP112 emulsion in sausage production might enhance the binding ability between protein, moisture and fat to reduce cooking loss.

Regarding proximate composition, ME100 samples showed the highest moisture (~67.19%), protein contents (~15.34%) and lowest fat content (~16.97%), while the control treatment (ME0) had the lowest moisture (~60.00%), protein contents (~13.26%) and highest fat content (~25.70%) (*p* < 0.05). By totally replacing pork back fat with MP112 emulsion, more than 30% fat reduction was achieved in ME100 samples compared with ME0 samples, which falls in the “reduced total fat content” category [39].

The replacement of animal fat with MP112 emulsion exhibited no effect on the ash content significantly. However, besides the ash content of sausages, other proximate composition of sausages showed a clear linear relationship related to the addition of MP112 emulsion. The fat content of emulsified sausages decreased linearly in response to the increased replacement ratio of MP112 emulsion (*p* < 0.001). The moisture and protein contents were inversely proportional to the fat content. Otherwise, the moisture and protein contents of the emulsified sausages increased linearly, corresponding to the increasing MP112 emulsion percentage (*p* < 0.001). The increasing moisture content may have a relationship with the higher moisture content in MP112 emulsion. The difference in the composition of MP112 emulsion compared to pork back fat resulted in the linear changes in the protein and fat content of emulsified sausages, perhaps due to the emulsion formulation with caseinate and mannoprotein. Similarly, linear relationships between the moisture and protein contents of sausages and the substitution rate of emulsion were also observed from the prepared low-fat emulsified sausage formulated with konjac glucomannan/oat β-glucan composite hydrogel [40]. As animal fat substitutes, the utilization of different emulsions as animal fat substitutes could be an efficient way to decrease the total fat content of different meat products by replacing fat with moisture.

The linear relationship of these compositions in sausages in response to the increase in MP112 emulsion indicated that the incorporation of MP112 emulsion as a fat replacer could lock the nutrient composition in the products, providing a potential enhancement to the nutritional value. This was not observed in other similar studies. For instance, when emulsion gels contained peanut oil, linseed oil and egg white powder, an increment of moisture and protein contents and a decrement of fat content of emulsified sausages formulated with emulsion gels was reported by Nacak et al. [1]. However, there was no significant linear relationship between these compositions of sausages and the substitution rate of emulsion gels. Kavuşan et al. [41] made fresh chicken sausages with gelled emulsion containing black cumin and flaxseed oil, whereas there were linear relationships between the chemical composition of uncooked fresh sausages and the level of gelled emulsion. After cooking, the chemical composition of cooked fresh sausages with different levels of gelled emulsion lost their linear relationships, accompanied by a decrement in water holding capacity.

### 3.3. Fatty Acids

Reducing animal fat and improving the fatty acid composition of meat products have become an emerging approach towards a healthier diet, since saturated fat is implicated in several diseases such as obesity and CHD. Hence, the effects of MP112 emulsion on fatty acid content were investigated. As shown in Table 4, the fatty acid composition of emulsified sausages from different treatments was analyzed from different treatments. As expected, significant differences were detected in the fatty acid composition of the sausages (*p* < 0.05) due to the replacement of animal fat with MP112 emulsion.

In all samples, palmitic acid (C16:0), stearic acid (C18:0), oleic acid (C18:1n9c) and linoleic acid (C18:2n6c) were the main components forming the fatty acid profile of the sausages. The contents of palmitic acid, stearic acid and oleic acid decreased linearly in response to the increasing percentage of MP112 emulsion, ranging within 9.33–23.74%, 4.39–10.97% and 27.55–44.54%, respectively, while the content of linoleic acid increased linearly from 13.58% (ME0) to 56.81% (ME100) (*p* < 0.001). Therefore, we deduced that the percentage of total PUFA increased linearly with increasing MP112 emulsion from 15.42% (ME0) to 62.86% (ME100) due to the higher linoleic acid content in MP112 emulsion prepared with olive oil blend (*p* < 0.001). In contrast, the percentage of total SFA and MUFA of sausages decreased linearly with increased MP112 emulsion, decreasing by 22.0% and 19.4%, respectively. Abraham et al. [42] reported that PUFA could reduce plasma cholesterol levels with lower blood pressure and prevent cardiac arrhythmias. Similar results were also observed from the inulin gelled emulsion, which was used as a fat replacer in Bologna sausage, resulting in increasing PUFA and decreasing SFA and MUFA content in the fatty acid composition [43].

The ratio of PUFA/SFA is an important nutritional parameter to judge nutritional value and healthiness of food products. In general, the PUFA/SFA ratio of whole diets should be higher than 0.45 [44]. In this study, the PUFA/SFA ratio linearly increased with the increase in MP112 emulsion, ranging from 0.32 (ME0) to 2.21 (ME100). When the replacement ratio of MP112 emulsion was up to 25%, the ratio of PUFA/SFA was identified within the recommended range. Ibrahim [45] emphasized that the high n-6/n-3 ratio in our diets could provoke adverse health impacts, and the recommended n-6/n-3 ratio was lower than 4 [46]. Although the ratio of n-6/n-3 in sausages decreased linearly with increased MP112 emulsion from 22.2% (ME0) to 11.64% (ME100), values higher than the recommendations were also observed (*p* < 0.01). These results suggest that MP112 emulsion could be good a fat substitute for meat products; it reduces the fat content of sausages and improves the nutritional composition of fatty acids. 

### 3.4. Lipid Oxidation during Processing

Besides microbial degradation, lipid oxidation is the major reason for quality deterioration during the storage of meat products; it forms toxic compounds which induce off-flavor, poor taste and loss of nutritional value. The degree of unsaturation in meat product is one of the main reasons for increasing oxidation susceptibility [47,48]. The TBARS values of sausages with different replacement ratios of MP112 emulsion during storage were determined. As shown in Table 5, the initial TBARS values of sausages with different treatments varied between 0.17 and 0.62 mg malondialdehyde (MDA)/kg meat.

In this study, the TBARS values of sausages decreased significantly (*p* < 0.01) in response to the increasing MP112 emulsion percentage. However, in other studies, using plant oil-based emulsion as a fat replacer could present a higher TBARS value due to the higher unsaturated fat content in vegetable oil. Müge et al. [49] studied the effects of replacing pork back fat with various vegetable oils, including olive oil, grape seed oil, corn oil, canola oil and soybean oil, and rice bran fiber on reduced-fat frankfurters, and the results showed that all plant oil samples exhibited significant higher TBA values of frankfurters than the control containing no added vegetable oil. These results indicated that the addition of MP112 emulsion could effectively inhibit lipid oxidation in sausages with high PUFA content.

Otherwise, the TBARS values of all samples increased throughout the storage, and the linear slope’s absolute values of TBARS values of sausages also increased with different treatments at the same storage time, suggesting that utilization of MP112 emulsion delayed lipid oxidation rate in sausages with excellent oxidative stability. In the food industry, efficient and low-cost synthetic antioxidants, such as sodium nitrite and butylated hydroxytoluene (BHT), are widely used in meat products to maintain meat quality and extend shelf-life. However, considering the potential health risk, natural compounds with broad antioxidant activity are increasingly demanded [50,51].

In recent studies, various natural antioxidants are applied in sausage formulations with effective antioxidant ability to delay lipid oxidation [12,52]. Polysaccharides and oligosaccharides with proper structural and functional properties are regarded as natural bio-preservatives and antioxidants [53]. The proteins and polysaccharide fraction, including β-glucans and mannans from yeast cell walls, exhibited excellent antioxidative capabilities, which were regarded as antioxidant polymers [54]. Mannan is the main component of mannoprotein MP112 polysaccharide, with a mannan to protein ratio of 14.5. Galinari et al. [55] obtained α-D-mannan (KMM-5) from the cell wall of *Kluyveromyces marxianus* CCT7735 using a centrifugal filter and demonstrated that KMM-5 exhibited an excellent antioxidant ability by chelating copper and iron ions. Indeed, these proteins extracted from yeast cell walls could be more antioxidative than these polysaccharides due to the presence of aromatic side chains and thiol groups. When evaluating the in vitro antioxidant potential of sparkling wines produced with β-glucanases, autolysated yeasts, yeast cell walls and purified mannoproteins, respectively, sparkling wines with the addition of purified mannoproteins exhibited the highest values of antioxidant activity [56]. Meanwhile, mannoprotein and β-glucan are proposed as the prebiotics with similar antimicrobial and prebiotic properties [57]. β-glucan has been proven to have a positive impact on the lipid oxidation and microbiological properties of sausages during storage periods, decreasing TBARS values and enhancing the growth of lactic acid bacteria [58,59]. The addition of MP112 emulsion could improve the nutritional composition of sausages and play an antioxidant role with prebiotic properties, which might be effective to delay meat spoilage and prolong the shelf life.

### 3.5. Textural Properties

Due to the important role of fat content in the texture, mouthfeel and bite of meat products, animal fat reduction accompanied by moisture increment might cause negative effects on texture and sensory quality features of meat products [60]. The incorporation of emulsions as fat replacers into formulations could result in a soft and sticky texture [41,61]. Hence, the texture properties of emulsified sausages with MP112 emulsion from different treatments was determined (Table 6), including hardness, springiness, cohesiveness and chewiness.

The results suggested that utilization of MP112 emulsion as a fat replacer in sausage formulation obviously altered the texture profile properties, and a significant improvement in the hardness, springiness, cohesiveness and chewiness of emulsified sausages was observed compared to the control (*p* < 0.05), indicating that MP112 emulsion could effectively improve the texture of emulsified sausages. The protein content of meat products is related to hardness, chewiness and gumminess. According to previous studies, when emulsions were prepared by pre-emulsification technique to replace fat, more meat proteins were available to bind water and form stable matrices; meanwhile, the addition of non-meat proteins also participated in the gel network [33]. The substitution of animal fat with a pre-emulsified canola oil system with plasma proteins was also reported to increase the hardness and chewiness of frankfurters [62]. Mannoprotein MP112 can be regarded as a protein–polysaccharide composite emulsifier due to the presence of protein and mannan, which can promote the texture and network structures in sausages. Otherwise, MP112 emulsion showed a pseudo plastic (non-Newtonian) behavior in the rheology test, which improved the gel strength and water holding capacity of myofibrillar protein composite gels [23]. Thus, in this study, with the addition of MP112 emulsion, more mannoproteins was available in the system to enhance the cross-linking with meat proteins and promote the formation of a well-structured gel, resulting in effective moisture retention and texture improvement in sausages.

In the meantime, with increased replacement ratio of MP112 emulsion, the springiness and cohesiveness of sausages also increased by degrees, while the hardness and chewiness showed a trend of decline after an initial ascent. ME50 and ME75 samples exhibited no significant difference in hardness and chewiness (*p* > 0.05); however, the hardness and chewiness of ME100 samples decreased significantly compared to ME75 samples (*p* < 0.05). The high moisture content of meat products could result in the formation of a softer structure [63], and chewiness was positively correlated with hardness [12]. Although the higher mannoprotein content of sausages had a more stable gel network with higher cohesiveness, excessive moisture content could increase the free water in sausages, resulting in a softer texture with lower hardness and chewiness.

### 3.6. Sensory Analysis

The sensory properties of sausage samples from different treatments were evaluated at day 0. As shown in Table 7, the appearance, flavor, juiciness and the overall acceptability of sausage samples exhibited no significant differences among sausages from different treatments (*p* > 0.05). These results implied that the application of MP112 emulsion could reduce fat without producing unfavorable changes in sensory parameters. Similar results were also observed by Burcu [30], when emulsified sausages were prepared with whey protein as a fat replacer. As the degree of substitution of MP112 emulsion increased to 50–75%, it was obvious that the ME50 and ME75 samples presented a higher texture score than the ME0 samples (*p* < 0.05), but the texture score was reduced in the ME100 samples compared with the ME75 samples, which is consistent with the changes to the textural properties in prepared sausages. Thus, emulsified sausages had better sensory attributes at a 50–75% replacement ratio of MP112 emulsion based on the sensory scores.

## 4. Conclusions

In summary, this study identified the potential application of MP112 emulsion as a fat replacer in sausage production. Replacing pork back fat by MP112 emulsion could markedly reduce fat content and improve the nutritional composition of emulsified sausages, resulting in lower total fat and saturated fatty acid contents and higher poly-unsaturated fatty acid content at low degrees of substitution. ME100 samples had better fatty acid compositions with the highest PUFA/SFA ratio and lowest n-6/n-3 ratio compared to control. Otherwise, addition of MP112 emulsion significantly delayed the rate of lipid oxidation in sausages during storage, especially at the level where fat is completely replaced. Meanwhile, the application of MP112 emulsion enhanced the network structure of sausages and improved their textural properties without having an uncomfortable impact on the general acceptance score compared with the control. Hence, using MP112 emulsion as a replacement of animal fat is regarded as a potential approach to prepare healthier meat products. In future investigations, it might be possible to assess the incorporation of emulsion with different emulsifier formulations containing different types of edible oils and mannoprotein MP112 to ensure the sensory, stability and oxidative quality of such products during storage.

## Figures and Tables

**Table 1:** Emulsified sausage formulation.

Sample	Replacement Ratio of Emulsion				
	0%	25%	50%	75%	100%
Pork leg muscle, g (%)	140 (64)	140 (64)	140 (64)	140 (64)	140 (64)
Back fat, g (%)	60 (24)	45 (18)	30 (12)	15 (6)	0 (0)
Water/ice, g (%)	45 (18)	45 (18)	45 (18)	45 (18)	45 (18)
Emulsion, g (%)	0 (0)	15 (6)	30 (12)	45 (18)	60 (24)
Salt, g (%)	3 (1.2)	3 (1.2)	3 (1.2)	3 (1.2)	3 (1.2)
Sugar, g (%)	1 (0.4)	1 (0.4)	1 (0.4)	1 (0.4)	1 (0.4)
Phosphate, g (%)	0.6 (0.24)	0.6 (0.24)	0.6 (0.24)	0.6 (0.24)	0.6 (0.24)
Pepper, g (%)	0.4 (0.16)	0.4 (0.16)	0.4 (0.16)	0.4 (0.16)	0.4 (0.16)
Total, g (%)	250 (100)	250 (100)	250 (100)	250 (100)	250 (100)

**Table 2:** Color of emulsified sausages prepared with MP112 emulsion.

Sample	L*	a*	b*
ME0	76.54 ± 1.84 ^c^	2.46 ± 0.17 ^a^	10.49 ± 0.55 ^a^
ME25	77.44 ± 0.30 ^b^	2.31 ± 0.04 ^a^	10.45 ± 0.11 ^a^
ME50	77.40 ± 0.32 ^b^	2.27 ± 0.10 ^ab^	10.37 ± 0.16 ^b^
ME75	78.28 ± 0.21 ^a^	2.20 ± 0.05 ^b^	10.13 ± 0.13 ^b^
ME100	78.56 ± 0.23 ^a^	2.07 ± 0.12 ^c^	10.07 ± 0.21 ^b^

The data were expressed as the mean ± SD in the table. Means with different superscript letters in the same column indicate significant differences (*p* < 0.05, *n* = 6).

**Table 3:** Cooking loss and proximate analysis of emulsified sausages containing MP112 emulsion.

Sample	Cooking Loss (%)	Moisture (%)	Protein (%)	Fat (%)	Ash (%)
ME0	2.18 ± 0.17 ^a^	60.00 ± 0.47 ^d^	13.26 ± 0.03 ^c^	25.70 ± 0.49 ^a^	2.09 ± 0.06 ^a^
ME25	1.97 ± 0.10 ^ab^	63.26 ± 0.66 ^c^	13.40 ± 0.11 ^c^	21.69 ± 0.27 ^b^	1.95 ± 0.18 ^a^
ME50	1.90 ± 0.07 ^b^	63.23 ± 0.67 ^c^	14.43 ± 0.17 ^b^	20.43 ± 0.19 ^c^	2.20 ± 0.10 ^a^
ME75	1.88 ± 0.06 ^b^	66.06 ± 0.88 ^b^	14.83 ± 0.06 ^b^	18.68 ± 0.06 ^d^	2.04 ± 0.04 ^a^
ME100	1.89 ± 0.11 ^b^	67.19 ± 0.57 ^a^	15.34 ± 0.12 ^a^	16.97 ± 0.64 ^e^	2.13 ± 0.02 ^a^
Linear effect ^‡^	ns	+ ^***^	+ ^***^	− ^***^	ns

^‡^ The + sign indicates a positive response to increasing percent MP112 emulsion, while the − sign indicates the opposite. ^‡^ Indicates the linear effect; ^***^: significant at the 0.001 level of probability, respectively; ns: not significant. The data were expressed as the mean ± SD in the table. Means with different superscript letters in the same column indicate significant differences (*p* < 0.05, *n* = 3).

**Table 4:** Fatty acid composition of emulsified sausages prepared with MP112 emulsion.

Fatty Acids	ME0	ME25	ME50	ME75	ME100	Linear Effect ^‡^
Saturated fatty acids						
C14:0	1.06 ± 0.02 ^a^	1.06 ± 0.05 ^a^	0.73 ± 0.03 ^b^	0.47 ± 0.03 ^c^	0.25 ± 0.00 ^d^	− ^**^
C15:0	0.31 ± 0.00 ^a^	0.20 ± 0.01 ^b^	0.18 ± 0.03 ^c^	0.15 ± 0.00 ^d^	0.03 ± 0.01 ^a^	− ^*^
C16:0	23.74 ± 0.18 ^a^	21.67 ± 0.34 ^b^	17.18 ± 0.02 ^c^	13.29 ± 0.26 ^d^	9.33 ± 0.28 ^e^	− ^***^
C17:0	0.46 ± 0.05 ^a^	0.30 ± 0.09 ^b^	0.18 ± 0.00 ^c^	0.17 ± 0.03 ^c^	0.15 ± 0.00 ^d^	− ^*^
C18:0	10.97 ± 0.28 ^a^	9.01 ± 0.18 ^b^	7.86 ± 0.34 ^c^	6.35 ± 0.23 ^d^	4.39 ± 0.52 ^e^	− ^***^
C20:0	0.20 ± 0.01 ^a^	0.15 ± 0.00 ^c^	0.18 ± 0.01 ^b^	0.22 ± 0.02 ^a^	0.18 ± 0.00 ^b^	ns
C21:0	0.90 ± 0.02 ^a^	0.71 ± 0.01 ^b^	0.58 ± 0.02 ^c^	0.42 ± 0.05 ^d^	0.20 ± 0.00 ^e^	− ^***^
C22:0	0.62 ± 0.02 ^a^	0.53 ± 0.03 ^b^	0.42 ± 0.00 ^c^	0.26 ± 0.01 ^d^	0.09 ± 0.01 ^e^	− ^**^
Monounsaturated fatty acids						
C15:1	0.20 ± 0.07 ^a^	0.14 ± 0.02 ^b^	0.16 ± 0.00 ^b^	0.05 ± 0.00 ^c^	0.06 ± 0.01 ^c^	− ^*^
C16:1	1.83 ± 0.09 ^b^	1.95 ± 0.08 ^a^	1.34 ± 0.01 ^c^	0.88 ± 0.02 ^d^	0.45 ± 0.02 ^e^	− ^*^
C17:1	0.18 ± 0.03 ^a^	0.18 ± 0.00 ^a^	0.12 ± 0.01 ^b^	0.11 ± 0.02 ^b^	0.07 ± 0.02 ^c^	− ^**^
C18:1n9t	0.17 ± 0.03 ^a^	0.15 ± 0.00 ^b^	0.17 ± 0.03 ^a^	0.12 ± 0.02 ^c^	0.10 ± 0.02 ^d^	ns
C18:1n9c	44.54 ± 0.20 ^a^	41.74 ± 0.12 ^b^	37.74 ± 0.18 ^c^	33.39 ± 0.27 ^d^	27.55 ± 0.73 ^e^	− ^***^
C20:1	0.90 ± 0.02 ^a^	0.71 ± 0.01 ^b^	0.58 ± 0.02 ^c^	0.42 ± 0.05 ^d^	0.20 ± 0.00 ^e^	− ^***^
Polyunsaturated fatty acids						
C18:2n6c	13.58 ± 0.46 ^e^	21.38 ± 0.38 ^d^	32.76 ± 0.33 ^c^	42.91 ± 0.49 ^b^	56.81 ± 0.63 ^a^	+ ^***^
C18:3n6	0.03 ± 0.00 ^d^	0.05 ± 0.01 ^c^	0.07 ± 0.02 ^c^	0.11 ± 0.00 ^b^	0.16 ± 0.01 ^a^	+ ^**^
C18:3n3	0.55 ± 0.05 ^e^	1.15 ± 0.03 ^d^	1.85 ± 0.02 ^c^	3.58 ± 0.32 ^b^	4.66 ± 0.23 ^a^	+ ^**^
C20:2	0.61 ± 0.02 ^a^	0.53 ± 0.03 ^b^	0.42 ± 0.00 ^c^	0.26 ± 0.01 ^d^	0.09 ± 0.00 ^e^	− ^**^
C20:3n6	0.08 ± 0.01 ^a^	0.07 ± 0.00 ^a^	0.08 ± 0.00 ^a^	0.04 ± 0.00 ^c^	0.06 ± 0.00 ^b^	ns
C20:3n3	0.08 ± 0.00 ^a^	0.06 ± 0.02 ^b^	0.05 ± 0.00 ^b^	0.04 ± 0.00 ^c^	0.03 ± 0.00 ^c^	− ^**^
C20:4n6	0.31 ± 0.06 ^a^	0.32 ± 0.05 ^b^	0.27 ± 0.01 ^c^	0.20 ± 0.02 ^d^	0.17 ± 0.00 ^d^	− ^*^
C22:2	0.17 ± 0.06 ^a^	0.07 ± 0.00 ^b^	0.06 ± 0.00 ^b^	0.05 ± 0.01 ^b^	0.07 ± 0.00 ^b^	ns
Sums and ratios of fatty acids						
∑SFA	36.64 ± 0.32 ^a^	32.32 ± 0.12 ^b^	26.36 ± 0.35 ^c^	20.82 ± 0.22 ^d^	14.67 ± 0.46 ^e^	− ^***^
∑MUFA	47.82 ± 0.32 ^a^	44.85 ± 0.25 ^b^	40.12 ± 0.03 ^c^	34.99 ± 0.31 ^d^	28.43 ± 0.55 ^e^	− ^***^
∑PUFA	15.42 ± 0.71 ^e^	23.96 ± 0.33 ^d^	36.73 ± 0.34 ^c^	47.17 ± 0.52 ^b^	62.86 ± 0.31 ^a^	+ ^***^
PUFA/SFA	0.32 ± 0.01 ^e^	0.53 ± 0.01 ^d^	0.92 ± 0.01 ^c^	1.35 ± 0.02 ^b^	2.21 ± 0.09 ^a^	+ ^**^
∑n-3	0.63 ± 0.02 ^e^	1.21 ± 0.01 ^d^	2.35 ± 0.02 ^c^	3.60 ± 0.04 ^b^	4.96 ± 0.09 ^a^	+ ^**^
∑n-6	14.00 ± 0.45 ^e^	22.15 ± 0.37 ^d^	33.90 ± 0.32 ^c^	43.26 ± 0.49 ^b^	57.74 ± 0.51 ^a^	+ ^***^
n-6/n-3	22.20 ± 0.51 ^a^	18.26 ± 0.27 ^b^	14.43 ± 0.92 ^c^	12.03 ± 0.11 ^d^	11.64 ± 0.10 ^e^	− ^**^

^‡^ The + sign indicates a positive response to increasing percent MP112 emulsion, while the − sign indicates the opposite. ^‡^ Indicates the linear effect; ^*^, ^**^, ^***^: significant at the 0.05, 0.01, 0.001 level of probability, respectively; ns: not significant. The data were expressed as the mean ± SD in the table. Means with different superscript letters in the same row indicate significant differences (*p* < 0.05, *n* = 3). ∑SFA = sum of saturated fatty acids; ∑MUFA = sum of monounsaturated fatty acids; ∑PUFA = sum of polyunsaturated fatty acids; ∑n6 = sum of omega-6 fatty acids; ∑n3 = sum of omega-3 fatty acids.

**Table 5:** Changes in thiobarbituric acid-reactive substances (TBARS) of emulsified sausages (mg MDA/kg).

Sample	0 d	3 d	7 d
ME0	0.62 ± 0.06 ^Aa^	0.93 ± 0.06 ^Ba^	1.98 ± 0.15 ^Ca^
ME25	0.54 ± 0.03 ^Ab^	0.73 ± 0.06 ^Bb^	1.35 ± 0.08 ^Cb^
ME50	0.40 ± 0.05 ^Ac^	0.53 ± 0.06 ^Bc^	1.06 ± 0.02 ^Cc^
ME75	0.38 ± 0.11 ^Ad^	0.49 ± 0.05 ^Ac^	0.91 ± 0.07 ^Bc^
ME100	0.17 ± 0.05 ^Ae^	0.28 ± 0.03 ^Bd^	0.56 ± 0.07 ^Cd^
Linear effect ^‡^	− ^**^	− ^**^	− ^**^
k	−0.424	−0.616	−1.312
R^2^	0.945	0.969	0.944

^‡^ The − sign indicates a negative response to increasing percent MP112 emulsion. ^‡^: The linear effect; ^**^: significant at the 0.01 level of probability, respectively; ns: not significant. The data were expressed as the mean ± SD in the table. Means with different superscript lowercase letters in the same column indicate the significant difference of sausages with different treatments at the same storage time; means with different superscript capital letters in the same row indicate the significant difference of the change in TBARS values in the same group during short-time storage (*p* < 0.05, *n* = 3). k: slope of the linear function; R^2^: coefficient of determination of the linear function.

**Table 6:** Texture profile of emulsified sausages prepared with MP112 emulsion.

Sample	Hardness (N)	Springiness	Cohesiveness	Chewiness (N‧mm)
ME0	37.33 ± 3.92 ^e^	0.83 ± 0.03 ^b^	0.56 ± 0.02 ^c^	21.71 ± 2.33 ^e^
ME25	42.24 ± 5.30 ^c^	0.86 ± 0.01 ^a^	0.59 ± 0.02 ^b^	26.88 ± 3.31 ^c^
ME50	48.92 ± 4.48 ^a^	0.86 ± 0.03 ^a^	0.60 ± 0.04 ^b^	30.28 ± 2.69 ^a^
ME75	47.95 ± 2.44 ^ab^	0.87 ± 0.02 ^a^	0.60 ± 0.03 ^b^	29.87 ± 2.54 ^ab^
ME100	40.13 ± 2.50 ^d^	0.88 ± 0.02 ^a^	0.69 ± 0.03 ^a^	22.97 ± 1.43 ^d^

The data were expressed as the mean ± SD in the table. Means with different superscript letters in the same column indicate significant differences (*p* < 0.05, *n* = 6).

**Table 7:** Sensory scores of emulsified sausages prepared with MP112 emulsion.

Sample	Appearance	Flavor	Texture	Juiciness	Overall Acceptability
ME0	7.60 ± 0.32 ^a^	7.65 ± 0.43 ^a^	6.72 ± 0.24 ^a^	7.02 ± 0.12 ^a^	7.41 ± 0.37 ^a^
ME25	7.53 ± 0.58 ^a^	7.63 ± 0.22 ^a^	7.05 ± 0.16 ^ab^	6.94 ± 0.24 ^a^	7.38 ± 0.22 ^a^
ME50	7.58 ± 0.24 ^a^	7.66 ± 0.16 ^a^	7.36 ± 0.19 ^b^	7.06 ± 0.52 ^a^	7.45 ± 0.24 ^a^
ME75	7.61 ± 0.17 ^a^	7.59 ± 0.11 ^a^	7.30 ± 0.26 ^b^	7.04 ± 0.44 ^a^	7.42 ± 0.16 ^a^
ME100	7.54 ± 0.61 ^a^	7.61 ± 0.29 ^a^	7.08 ± 0.15 ^ab^	7.13 ± 0.24 ^a^	7.35 ± 0.43 ^a^

The data were expressed as the mean ± SD in the table. Means with different superscript letters in the same column indicate significant differences (*p* < 0.05, *n* = 12).

## Data Availability

The data used to support the findings of this study can be made available by the corresponding author upon request.

## Supplementary Materials

The following supporting information can be downloaded at: https://www.mdpi.com/article/10.3390/foods12132486/s1, Figure S1: The enzymatic preparation of MP112 from baker’s yeast by β-1,6-glucanase (the red diagram) [26].
