# Supplementary material for: Quality Characteristics of Reduced-Fat Emulsified Sausages Made with Yeast Mannoprotein Enzymatically Prepared with a β-1,6-glucanase
Source: Foods. 2023 Jun 26;12(13):2486. doi: 10.3390/foods12132486 (PMC10341360; doi:10.3390/foods12132486)
Supplement: Supplementary file 1 [file foods-12-02486-s001.zip › foods-2436960-supplementary.pdf]

Quality Characteristics of Reduced-Fat Emulsified Sausages Made with Yeast Mannoprotein Enzymatically Prepared with a β-1,6-glucanase

Supplementary Materials

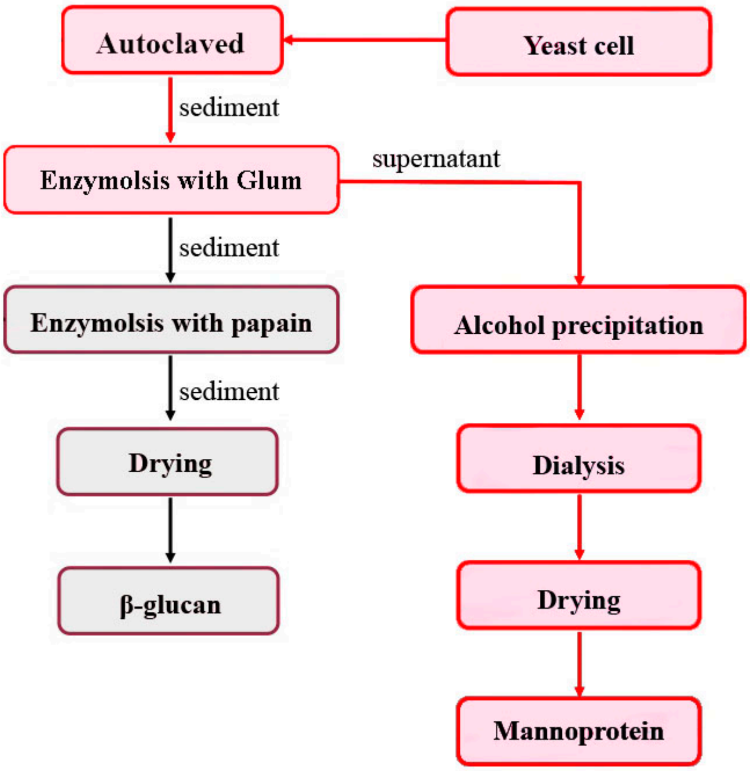

Figure S1. The enzymatic preparation of MP112 from baker's yeast by β-1,6-glucanase (the red diagram) [26].

26. Qiao, Y.; Ye, X.; Zhong, L.; Xia, C.; Zhang, L.; Yang, F.; Li, Y.; Fang, X.; Fu, L.; Huang, Y.; et al. Yeast β-1,3-glucan production by an outer membrane β-1,6-glucanase: Process optimization, structural characterization and immunomodulatory activity. *Food Funct.* **2022**, *13*, 3917–3930. https://doi.org/10.1039/d1fo02832d
